# Breast carcinoma and malignant melanoma metastasis within a single axillary lymph node

**DOI:** 10.1186/1477-7800-3-32

**Published:** 2006-10-06

**Authors:** Kirstin A Carswell, Kasim A Behranwala, Ashutosh Nerurkar A, Gerald PH Gui

**Affiliations:** 1 Breast Surgery Department, Royal Marsden Hospital, London, UK; 2 Pathology Department, Royal Marsden Hospital, London, UK; 3 Academic Surgery (Breast Unit), Royal Marsden Hospital, London SW3 6JJ, UK

## Abstract

A 58 year old lady presented with a right breast cancer and a prior history of malignant melanoma excised from the right chest wall eight years previously. An abnormal axillary lymph node resected contained features of both metastatic breast carcinoma and malignant melanoma. Following oncologic breast cancer management, the patient is well with no evidence of recurrence at three years.

## Background

There is an increased incidence of breast cancer as a second primary tumour in women previously diagnosed with a malignant melanoma [1]. Any direct association is at best controversial [2] and common aetiogical factors remain elusive. One plausible link may be hormonal in origin as oestrogen receptors are expressed in up to half of malignant melanomas [3] and at least two-thirds of breast cancers.

There are isolated case reports of melanoma and carcinoma found together in the primary tumour mass in breast [4], lung [5], oral cavity [6,7]. Cancer-to-cancer metastasis with evidence of melanoma metastases to primary renal cell carcinoma [8] is also documented. There is only one report of synchronous involvement in the metastatic axillary lymph node retaining the dual histology of the breast tumour exhibiting melanoma and carcinoma components [4]. We report on a lymph node containing two different primary cancer types that may result in distinct clinical management and have different prognostic implications for the patient.

## Case report

**Figure 1:**
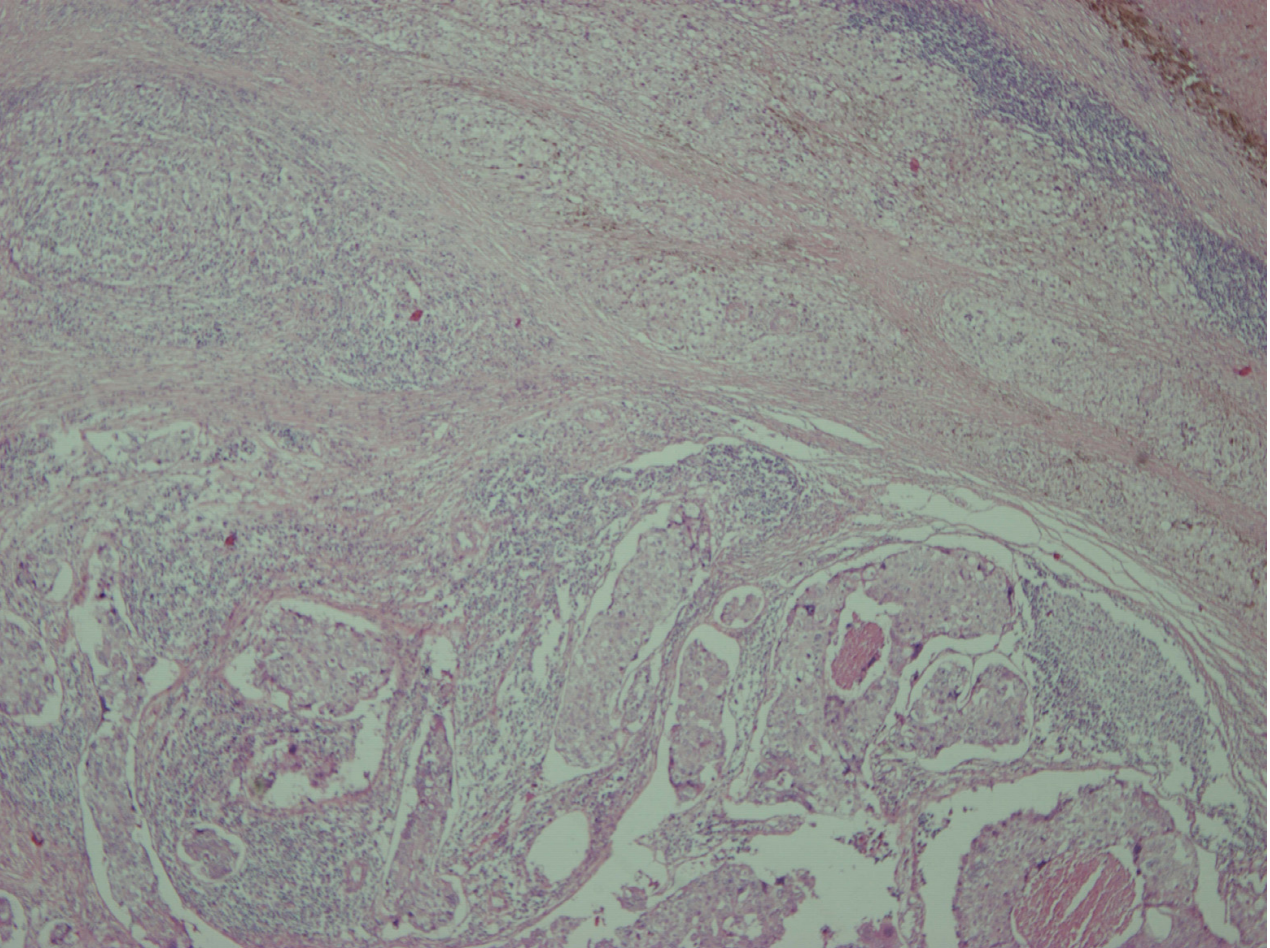
Metastasis of melanoma and carcinoma to a single regional lymph node, the upper part shows melanoma while lower part of photo shows carcinoma (40×).

**Figure 2:**
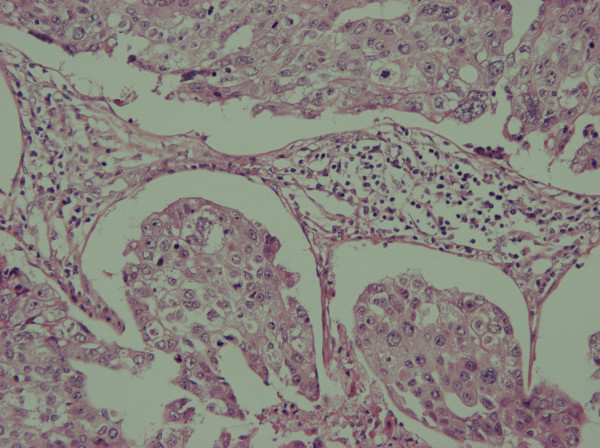
Carcinoma component (200×).

**Figure 3:**
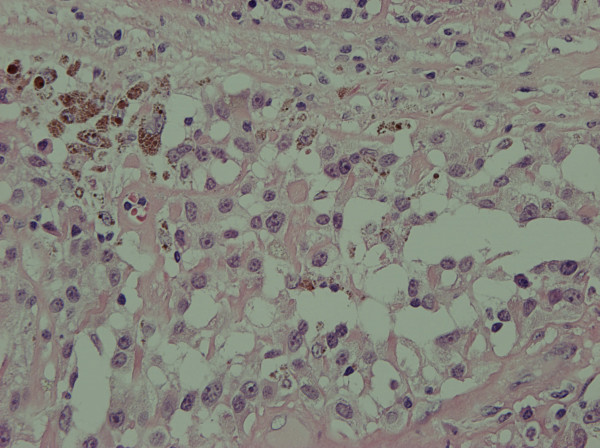
Melanoma component (400×).

A 58 year old lady presented with a 2.5 cm right breast lump inferior to the nipple with skin tethering and a palpable malignant right axillary lymph node. She had excision of a right chest wall malignant melanoma Clark's level IV (1.18 mm maximum thickness) eight years prior. There were no palpable axillary nodes at that time. Mammogram, ultrasound and fine needle aspiration cytology of the breast lump confirmed breast cancer. Core biopsy of the right axillary lymph node showed high grade malignancy including pigmented cells, possibly originating from the malignant melanoma. She underwent right wide local excision and axillary nodal clearance. Histopathology revealed a 19 mm, grade 3 infiltrating ductal carcinoma of the breast of medullary phenotype with no lymphovascular invasion that was ER, PgR and HER-2 negative. Breast tumour cells in the primary cancer were positive for CAM 5.2, AE1, AE3 and EMA and negative for S100 and HMB45. Right axillary nodal clearance showed one out of nine lymph nodes contained metastatic cancer with a large area of necrosis and 2 distinct tumour types (Figure 1). One morphological type was of epithelial origin with cells arranged in cohesive sheets. These areas were positive for CAM 5.2, AE1, AE3 and EMA (Figure 2). Other areas were highly pleomorphic containing cells with prominent nucleoli arranged in loose dissociate clusters that were strongly positive for S100 protein and focally for HMB45; negative for EMA and CAM 5.2 (Figure 3). The lymph node metastases therefore contained features of metastatic breast carcinoma and malignant melanoma in the same metastatic focus. Staging CT of her thorax, abdomen and pelvis as well as a bone scan were negative for distant spread. The patient was treated with adjuvant chemotherapy for breast cancer with 5 fluorouracil, epirubicin and cyclophosphamide for six cycles followed by radiotherapy to the breast. She remains well with no evidence of breast cancer or melanoma recurrence three years later.

## Discussion

Early diagnosis and better treatment protocols have led to significant improvements in disease free and overall survival following a diagnosis of cancer. It is therefore becoming increasingly common for patients to be diagnosed with a second primary tumour in their lifetime, particularly in patients with primary malignant melanoma [1,2]. The possible explanations include familial factors, the increasing natural lifespan of patients, and improved outcome following previous cancer treatment.

The primary breast cancer in our case did not contain melanoma cells that might have arisen through collision between two histologically different primary tumours, nor was there any pathological evidence of metaplasia, neuroendocrine differentiation or cancer to cancer metastases. The presence of two metastatic cell phenotypes in the axillary lymph node with different immunohistochemistry profiles is likely to have occurred in a metachronous fashion with the melanoma metastasis being detected incidentally during axillary dissection for breast carcinoma. The involved lymph node in probability represented the true sentinel node draining the skin of the chest wall and the breast. It is possible that melanoma micrometastases was dormant in the axillary lymph node and did not progress to clinical significance following the melanoma diagnosis. Melanoma micrometastases may therefore not be prognostically equivalent to overt nodal metastases and may reflect the natural history of melanoma micrometastases in the sentinel lymph node [9]. Barnhill et al [10] demonstrated that melanoma micrometastases lack significant rates of proliferation, apoptosis and neo-vascularisation when compared with macrometastases as the angiogenic and lymphangiogenic factors may be suppressed by host immune responses.

Subsequent management and follow-up of patients such as ours depend on the competing risks and natural history of the two separate primary cancers. In this case, adjuvant breast cancer treatment was of priority with continued surveillance of the melanoma.

The common sentinel node draining the breast and the trunk skin probably reflects the dominant lymphatic pattern of ectodermal structures and adds to the anatomical evidence to support the concept of the first draining node to a regional basin. The application of new technologies to cancer staging has to be guided by the natural history of the various cancer types. Detection of regional metastases has different prognostic implications for breast cancer and melanoma and management decisions need to be made on evidence based clinical criteria rather than any incidental findings.
